# Evaluation of the ABL NGS assay for HIV-1 drug resistance testing

**DOI:** 10.1016/j.heliyon.2023.e22210

**Published:** 2023-11-10

**Authors:** Thomas Lhossein, Karine Sylvain, Véronique Descamps, Virginie Morel, Baptiste Demey, Etienne Brochot

**Affiliations:** a Department of Virology, Amiens University Medical Center, Amiens, France; b Agents Infectieux Résistance et Chimiothérapie Research Unit, UR4294, Jules Verne University of Picardie, Amiens, France

**Keywords:** HIV, Resistance mutations, Drug resistance testing

## Abstract

HIV evolution and variability around the world requires special monitoring of the viral strains in infected people. High-throughput HIV sequencing and drug resistance testing techniques have become routinely available over the last few years.

We conducted a study to assess the new CE-marked ABL NGS HIV genotyping assay on an Illumina® platform, to compare the results (the detection of resistance associated mutations (RAMs) detected in the three main targets: reverse transcriptase, protease, and integrase) with those produced by three Sanger-based assays, and to compare the assays’ respective costs.

For the 10 samples and a 20 % sensitivity threshold for the NGS technology, the percent agreement between the four assays ranged from 99.5 % to 100 %. We detected 4 more and 10 more RAMs of interest when we lowered the NGS assay's threshold to 10 % and 3 %, respectively. At a threshold of 3 %, the antiretroviral sensitivity interpretation algorithm (for protease inhibitors) was modified for only two patients. The NGS assay's unit cost fell rapidly as the number of samples per run increased.

Compared with Sanger sequencing, the ABL NGS HIV genotyping assay is just as robust and somewhat more expensive but opens up interesting multiplexing perspectives for virology laboratories.

## 1 Introduction

Two retroviruses have been identified as agents of acquired immunodeficiency in humans. The human immunodeficiency viruses 1 and 2 (HIV-1 and HIV-2) resulted from several interspecies transmissions of simian viruses to humans [1]. To date, 95 circulating recombinant forms have been identified. HIV-1 and HIV-2 have been responsible for epidemics, such as those seen in south-east Asia [2,3]. HIV's resistance to various drug molecules can be assessed by phenotypic or genotypic testing. Phenotypic testing is reserved for highly specialized HIV research laboratories or require expertise, whereas genotypic tests are commonly used in laboratories monitoring infected patients [4,5]. In genotypic testing, the target genetic material is amplified in PCRs and the region of interest is then sequenced for mutations known to confer resistance to antiretrovirals. Testing focused on mutations in three viral genes (encoding HIV's reverse transcriptase (RT), protease and integrase) on which selective pressure is exerted by today's antiretroviral drugs. The protease is a 10 kDa protein composed of 99 amino acids [6]. It is fully active as a dimer. Via an autocatalytic reaction, it is cleaved from precursor proteins during the virus assembly process. RT is a very versatile RNA- and DNA-dependent DNA polymerase that synthesizes DNA copies from RNA [7]. The 32 kDa integrase (composed of 288 amino acids) is part of a catalytic complex that binds viral double-stranded DNA and integrates it into the host cell genome [8]. Genome-based resistance testing (i.e. genotyping) is a key step in the HIV treatment strategy. In several clinical situations, samples are screened for mutations in the genes coding for RT, protease, integrase, and sometimes glycoprotein 120 [9]. The guidelines are the same for adults and children: testing for resistance associated mutations (RAMs) is recommended in primary infections, before treatment initiation or in the event of virological failure. The current testing approach in clinical practice is based on Sanger sequencing. However, this has a number of technical limitations, such as the 20 % detection threshold. Thus, the identification of minority mutations is not feasible. Despite a number of technical and logistical difficulties, new high-throughput next-generation sequencing (NGS) technologies are becoming more common and are revolutionizing the field of microbiology. In principal, NGS technologies enable the more sensitive detection of RAMs and also open up opportunities in research [10,11]. Driven partly by the COVID-19 pandemic, many virology laboratories worldwide have purchased and implemented new NGS sequencing techniques. However, very few CE-marked NGS-based HIV-1 drug resistance testing assays are available [12]. The objectives of the present study were to evaluate (on an Illumina® platform) the CE-marked NGS version of the DeepChek® assay developed by the company ABL SA (Luxembourg City, Luxembourg), to compare the results (the detection of RAMs in the three main targets: RT, protease, and integrase) with those produced by three Sanger-based genotyping assays (Viroseq® from Abbott Molecular, which has been used as a reference assay in our laboratory for several years), the ABL DeepChek® assay in a Sanger sequencing format, and an in-house assay developed by the French national research agency (ANRS), and to compare the assays' respective unit costs.

## 2 Methods

### 2.1 Study design and cohort

The present study was conducted at the Amiens University Medical Center (Amiens, France). The samples of plasma used in the study had been sent to the virology laboratory as part of routine patient care. This study was conducted in accordance with the guidelines of the Amiens University Medical Center following approval by its institutional review board (PI2023_843_0006) with the principle of non-opposition contained in the information booklet.

To compare the assays’ results and avoid technical problems, two sample conditions were selected: a minimum viral load of 3 log_10_ copies/mL viral load (Roche Cobas® 4800), and a variety of mutation profiles. The selected plasma extracts had to be recent enough (<6 months) to limit RNA degradation and thus avoid biasing the assays. The samples and their characteristics are detailed in Table 1.

**Table 1:** Samples analyzed, RAMs, and viral load profiles.

Sample	Viral load (copy log)		Viroseq® Sanger resistance profile	ABL NGS mutations found (threshold: 3 %)		
		Subtype		Protease	Reverse transcriptase	Integrase
**1**	4.61	02_AG	PIs	16E, 36I, 69K, 89 M	No mutations	
**2**	4.68	02_AG	PIs	16E, 36I, 69K, 89 M	No mutations	
**3**	Unknown	02_AG	NRTIs + NNRTIs + PIs	36I, 69K, 89 M	181C, 184V, 221Y	74I
**4**	4.15	C	PIs	10I, 16E, 20R, 20 M, 36I, 63P, 69R, 69K, 89 M	90I	No mutations
**5**	6.24	B	No resistance	63P, 71V	No mutation	
**6**	5.52	B	No resistance	36I, 63P, 89 M	90I	No mutations
**7**	4.7	B	No resistance	60E,	69S	No mutations
**8**	5.8	02_AG	PIs	10V, 16E, 36I, 63P, 69K, 89 M	69 N, 106I	No mutations
**9**	5.11	02_AG	PIs	16E, 36I, 69K, 89 M	No mutation	74I
**10**	6.00	A1	PIs	36I, 69K, 89 M	179I	No mutations

PIs: protease inhibitors; NRTIs: nucleoside reverse transcriptase inhibitors; NNRTIs: non-nucleoside reverse transcriptase inhibitors.

## 3 Methods

RNA was extracted from 400 μL of plasma on the eMAG® system. Two extracts were made and compiled in order to carry out all the tests. This step was followed by RT-PCRs of the three target regions (RT, protease, and integrase). The ABL NGS assay, the ABL Sanger assay and the Viroseq™ Sanger assay (combining the reverse transcriptase and protease kit and ViroSeqHIV-1 Integrase) were performed according to the respective manufacturers' instructions. The primers and protocols for the ANRS Sanger assay are described at https://hivfrenchresistance.org/wp-content/uploads/2021/10/ANRS-procedures.pdf.

The various DNAs were purified with magnetic beads and quantified with the Qubit® kit. The pooled library was prepared according to standard procedures with a protocol provided by ABL. Qubit dsDNA HS assay was used for library quantification. The sequencing steps followed the Illumina® protocol using Miseq Reagent Kit v2 300 cycles. Applied Biosystems 3130xl was used as Sanger sequencer.

HIV-1 subtypes are determined as the best matches obtained by the BLAST search based on the RT sequence obtained with the Viroseq method.

### 3.1 Data analysis

The NGS results (in Fastq format) were analyzed on ABL's DeepChek® pipeline. The acceptable depth of coverage was 1000 reads for each region of the viral genome analyzed. The French algorithm (ANRS v32) was used for genotyping and for classifying and grouping all the resistance associated mutations, i.e. those that can confer resistance to one or more antiretroviral drugs for the four assays. SmartGene software has an integrated analysis for HIV that provides resistance interpretations of the resulting fasta sequences. The percent agreement and Cohen's kappa were calculated using GraphPad software.

## 4 Results

**Fig. 1:**
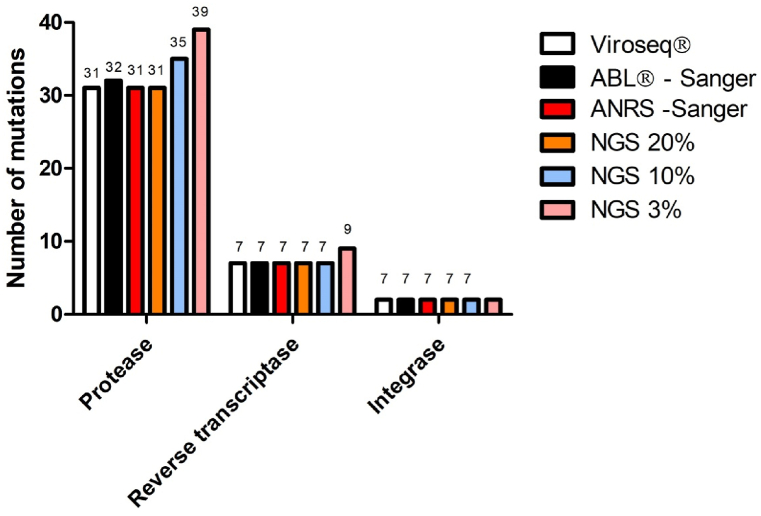
RAMs for the 10 available samples, according to the assay used.

The numbers of RAMs of interest detected by NGS and Sanger assays:

For the 10 samples analyzed, we counted and compared the RAMSs detected by the four genotyping methods (Fig. 1). More RAMs were detected when the NGS threshold was lowered. When considering the 10 samples evaluated with all four assays, we detected 4 more and 8 more RAMs in the protease gene at thresholds of 10 % and 3 %, respectively. For the RT region, we found only 2 additional RAMs (9 at the 3 % threshold, vs. 7 at the 10 % and 20 % thresholds). The 10 additional RAMs found with the 3 % NGS threshold affected the interpretation of the results with the ANRS algorithm for only two samples (samples 4 and 6: Table 2). With the 3 % threshold, patient 4's classification changed from “sensitive” to “intermediate” for the protease inhibitors atazanavir and lopinavir and from “intermediate” to “resistant” for tipranavir. Patient 6's classification changed from “sensitive” to “intermediate” for tipranavir.

**Table 2:** Impact of the sensitivity of molecules by detecting RAMs <20 %.

Samples and molecules concerned		Antiretroviral drug class	Interpretation of the resistance result, by threshold		Mutations affecting interpretation (%)
			Viroseq®, ABL® Sanger, ANRS, NGS (20 %)	NGS (3 %)	
**1**	Tipranavir/r	PIs	R	R	PR: **16E (3.1),** 36I (99.04), 69K (96.42), 89 M (98.4)
**2**	Tipranavir/r	PIs	R	R	PR: 16E (95.02), 36I (97.16), 69K (95.16), 89 M (98.99)
**3**	Abacavir	NRTIs	I	I	RT: 181C (97.75), 184V (64), 221Y (74.08)
	Emtricitabine lamivudine	NRTIs	R	R	
	Doravirine	NNRTIs	I	I	
	Efavirenz, etravirine, islatavir, nevirapine, rilpivirine	NNRTIs	R	R	
	Tipranavir/r	PIs	R	R	PR: 36I (98.82), 69K (98.23), 89 M (98.94)
**4**	Atazanavir/r	PIs	S	**I**	PR: **10I (10.28)**
	Lopinavir/r	PIs	S	**I**	PR: **10I (10.28), 20M (10.38),** 20R (68.85), **63P (10.3)**
	Tipranavir/r	PIs	I	**R**	PR: **69R (7.42),** 69K (81.31), **89M (11.42)**
**5**	Sensitive to all molecules				
**6**	Tipranavir/r	PIs	S	**I**	PR: **36I (3.14), 89M (3.14)**
**7**	Sensitive to all molecules				
**8**	Atazanavir/r	PIs	I	I	PR: 10V (42.65), 16E (99.09)
	Tipranavir/r	PIs	R	R	PR**:** 36I (99.4), 69K (99.09), 89 M (99.19)
**9**	Tipranavir/r	PIs	R	R	PR: 16E (98.9), 69K (98.7), 89 M (98.21)
**10**	Tipranavir/r	PIs	R	R	PR: 36I (99.76), 69K (99.43), 89 M (99.66)

PIs: protease inhibitors; NRTIs: nucleoside reverse transcriptase inhibitors; NNRTIs: non-nucleoside reverse transcriptase inhibitors; PR: protease; RT: reverse transcriptase; S: sensitive; I: intermediate; R: resistant.

The additional mutation detected by ABL NGS compared with the other Sanger assay are indicated in bold.

### 4.1 Level of agreement between the assays

We next calculated the level of agreement between the 4 genotyping assays by taking the NGS technique with a threshold of 3 % as the reference for our 10 samples. At the 21 positions where we obtained RAMs, we detected 50 RAMs with the NGS 3 % assay, 41 with the ABL Sanger assay and 40 for each of the NGS 20 %, Viroseq and ANRS assays (Table 3). The percent agreement ranged from 95.2 % to 100 % (Table 3A). Cohen's kappa for pairwise comparisons of the assays ranged from 0.867 to 1, which indicated almost perfect agreement between the assays (Table 3B).

**Table 3:** Agreement between the assays. Pairwise comparisons of the overall percent agreement for RAMs (A), and calculation of Cohen's kappa (B).

(A) Percent agreement								
n = 50	NGS 3 %		NGS 20 %		Viroseq® Sanger			ABL Sanger
NGS 3 %								
NGS 20 %	95.2							
Viroseq® Sanger	95.2		100					
ABL Sanger	95.7		99.5		99.5			
ANRS Sanger	95.2		100		100			99.5
(B) Cohen's kappa								
n = 50		NGS 3 %		NGS 20 %		Viroseq® Sanger	ABL® Sanger	
NGS 3 %								
NGS 20 %		0.867						
Viroseq® Sanger		0.867		1				
ABL Sanger		0.866		0.985		0.985		
ANRS Sanger		0.867		0.985		1	0.985	

### 4.2 Approximate costs for each technique

We next attempted to estimate the undiscounted purchase price per sample or per series of 5 or 20 samples for each kit or set of materials. The cost of any interpretation software required was not included in the calculation but is indicated at the bottom of Table 4. Likewise, we did not take into account of all the standard laboratory materials and consumables (such as multichannel pipettes, pipette cones, gels, and 96-well magnetic plates which was estimated to be between 5 and 10 euros/sample depending on the size of the sample set.) or labor. The price of the various automats is not mentioned here (Table 4). For NGS, the undiscounted price for a single sample was calculated so that a single sequencing cartridge could be completed. The choice of 5 patients per series reflects the routine level of demand in our laboratory. Unsurprisingly, we found that the in-house ANRS assay was the cheapest. The price of the Sanger assays depended on the price of the kits and the software license. By combining many samples in the same cartridge, the cost of the NGS assay falls as the number of samples in the run increases: from 506 euros per sample for a single sample to 189 euros per sample for a series of 20 samples (3783 euros/20).

**Table 4:** Undiscounted price per sample, and estimation based on possible discounts for the main reagents and materials.

Technique	Viroseq® Sanger	ABL® Sanger	NGS	ANRS Sanger
Materials and prices (1 sample)	Kit RT PROT € 225.15 Kit INT € 256.98 (Exosap included)	Kit RT PROT € 69.04 Kit INT € 50.21 Sequencing V2Purif € 25.66	Kit RT PROT € 69.04 Kit INT € 50.21 Nano Kit V2 € 334 Beads € 5.32 Qubit® € 2.06 DeepChek® Assay NGS Library preparation € 45.83 Bioanalyzer® (BioA) € 57	Primers € 3.60 RT PCR Qiagen® One step – € 13.8 Nested PCR - € 3.81 Purif exosap - € 3.09 Big Dye Terminator – € 43.50 X Terminator - € 36.27
Gross cost for 1 sample	**€ 482.12**	**€ 144.92**	**€ 506.46** **-** **€ 563.46** (with BioA)	**€ 104.07**
Series of 5 samples	**€ 2410.60**	**€ 724.60**	**€ 1196.30** **€ 1481.30 €** (with BioA)	**€ 520.35**
Series of 20 samples	**€ 9642.40**	**€ 2898.40**	**€ 3783.20** **€ 4923.20** (with BioA)	**€ 10407.30**
Software	Smartgene®**:** **€ 9500/an**	Viroscore®: **€ 6990**/year	DeepCheck®: **€ 6990**/year	Smartgene®**: € 9500/year**

BioA: Bioanalizer®.

## 5 Discussion

In order to evaluate a new technique for use in the virology laboratory, we studied the ABL NGS genotypic drug resistance assay and three Sanger-based assays: the currently used Viroseq kit from Abbott (which is discontinued by the manufacturer), ABL's Sanger kit, and an in-house technique including the ANRS primers. Each assay had strengths and weaknesses, as summarized below.

The most important variable is the reliable detection of RAMs. Here, Cohen's kappa highlighted the high overall level of agreement between the techniques with an NGS threshold of 20 % (κ >98.5 %) or 3 % (κ>86.7 %) (Table 3). The differences in the numbers of RAMs were small, and it is important to note that these differences did not influence the overall interpretation of antiretroviral resistance for each assay studied. The value of NGS lies in its ability to detect RAMs at thresholds <20 %. In our study samples, the NGS assay detected 7 more RAMs for protease and 2 more for RT (Fig. 1). It should be noted that the threshold that guarantees a 99 % confidence level is 3 %; below this threshold, the thousands of mutations detected correspond to background noise. Another point of NGS technology that could make it less useful than the Sanger technique is its ability to analyze samples with a viral load <3 log_10_ copies/mL. Since this evaluation, we have sequenced samples with low viral loads (around 2 log_10_ copies/mL) by this technique with good success rates.

We also observed that the detection of RAMs of interest below the 20 % threshold can change the interpretation of antiretroviral resistance (Table 2), as observed for only 2 samples in our series. However, there are still very few literature data on the possible clinical impact of these changes; it might be interesting to study the genome before initiating treatment for patients who have failed to respond to treatment, in order to determine whether lowering this threshold could be justified [13,14].

The Viroseq® kit had the advantage of having several pairs of overlapping primers, which is an excellent approach in reporting mutations. This aspect of sequencing is not at all a problem for NGS because of the technology used in the “clustering” step. Indeed, it even provides quantitative information on all the detected mutations. Nevertheless, the ABL NGS assay has some limitations. For example, it does not cover amino acid positions 319 to 348 for RT and positions <45 and >284 for integrase. However, it should be noted that no RAMs occur at these positions.

Regarding the price differences between assays (Table 4), it is advisable to choose an economical solution or at least one with a good price/performance ratio. It is also important to choose a technique that is CE-marked and is accompanied by simple, intuitive software. The cheapest assay per unit is undoubtedly the “in-house” sequencing assay with ANRS primers. However, the lack of optimal coverage (due to non-overlapping sequences) means that the results may be less accurate and of lower quality.

The NGS assay had the highest per sample price of the four assays; this is because (i) the price of a cartridge is fixed and so (ii) the number of samples to be sequenced affects the price. Hence, working with large series is more attractive for laboratories. Our example of a 20-patient series shows that the NGS assay can be much more cost-effective than some Sanger assays. The NGS assay's advantages this include the quantification of mutations, more sensitive detection, and the application of various criteria and quality filters guaranteeing a 99 % confidence level; this constitutes a very interesting solution in terms of performance and cost. The Bioanalyzer® is not required but is recommended (at least immediately after implementation of the method) to check the quality of the library, which increases the price. However, various strategies can be applied to increase the number of samples, such as the analysis of different genetic materials on the same cartridge: SARS-CoV-2, HBV, HCV, CMV, HSV, HPV, RNA16S, and resistance in tuberculosis [15]. The sequences of the 3 HIV assay targets account for a very small and almost negligible proportion of the cartridge's total capacity (0.5 GB). By way of an example, we could analyze 96 patients on a single cartridge (Nano V2). Furthermore, NGS assays have some logistical limitations: they take much longer to run than Sanger assays (2.5 days of technical work-up before sequencing can start with ABL and 2 days with the VELA Sentosa SQ HIV-1 Genotyping System [16]) and require specific training for operators. The automation of library preparation and other steps would minimize human intervention, reduce technician costs, increase reproducibility and productivity, and thus make the technique more attractive generally.

The limitations of our study include the small number of samples and the fact that we did not evaluate samples with a low viral load. On this point, after more than a year's use of NGS technology in our diagnostic laboratory, we have a success rate of over 84 % for the 3 regions of interest on viral loads of between 2 and 3 log_10_ copies/mL (38/45). For all samples above this threshold, we were able to perform the analysis.

Today, NGS assays and their perpetual technological evolution are making an undeniable contribution to the fields of oncology and genetic diseases. Hence, NGS techniques might be of value in microbiology [17]. High-throughput “shotgun” sequencing (i.e. a general metagenomics approach to sequencing all the nucleic acids in a given sample) has revolutionized the field [18,19]. From a technological point of view, shotgun sequencing removes the need for target-specific PCR primers. The process involves fragmentation enzymes, library construction and massive sequencing. In routine practice, a *de novo* sequencing approach might be of epidemiological interest for the detection of emerging viruses or the monitoring of endemic areas. Some researchers have even suggested that shotgun sequencing can save time in patient management by simplifying laboratory management.

The role of NGS technologies in patient care and personalized medicine has yet to be defined in international guidelines. With regard to urgent cases, it should be noted that at least 48–72 h are required for read generation. Nevertheless, NGS technologies undoubtedly represent a revolution in the fields of microbiology and infectious diseases as a whole. Compared with Sanger sequencing, the ABL NGS HIV genotyping assay is just as robust and somewhat more expensive but opens up interesting multiplexing perspectives for virology laboratories.

## Funding

This research did not receive any specific funding from agencies or organizations in the public, commercial, or not-for-profit sectors.

## Data availability statement

Data will be made available on request.

## CRediT authorship contribution statement

**Thomas Lhossein:** Writing – original draft, Validation, Investigation. **Karine Sylvain:** Validation, Methodology, Investigation, Data curation. **Véronique Descamps:** Validation, Investigation. **Virginie Morel:** Supervision, Investigation, Data curation. **Baptiste Demey:** Writing – review & editing, Conceptualization. **Etienne Brochot:** Writing – review & editing, Writing – original draft, Supervision, Project administration, Investigation, Conceptualization.

## Declaration of competing interest

The authors declare that they have no known competing financial interests or personal relationships that could have appeared to influence the work reported in this paper.
